# 2313 Characterization of the host pericyte role in glioblastoma angiogenesis

**DOI:** 10.1017/cts.2018.36

**Published:** 2018-11-21

**Authors:** Frank Attenello, Frank Attenello, Yingxi Wu, Kathleen Tsung, William Mack, Thomas Chen, Berislav Zlokovic

**Affiliations:** 1 University of Southern California; 2 Department of Neurosurgery, Keck School of Medicine of USC

## Abstract

OBJECTIVES/SPECIFIC AIMS: Glioblastoma (GBM) carries a prognosis of 14.6 months mean survival despite maximal surgical, chemotherapeutic, and radiation therapy. The pericyte is a recently characterized cell shown to be a critical component of cerebral vessel physiology and pathology. Importantly, alterations in pericyte densities have shown resulting changes in breast and lung tumor growth. We leverage transgenic pericyte-deficient mouse models to evaluate resulting behavior of implanted patient-derived GBM. METHODS/STUDY POPULATION: Patient-derived, green fluorescent protein labeled, GBM will be implanted in right frontal bregma of both 6-month old pericyte-deficient (PDGFR+/−) mice and age-matched wild-type littermate controls (IACUC 20755, IRB 16-00929), which are immunosuppressed via daily intraperitoneal cyclosporine injection. In total, 30 mice of both genders are included in tumor and control cohorts. Fixed cortical sections following 3-week period will be stained for pericytes (NG2), endothelium (CD31), hypoxia (piminidazole), and tumor size. One-way ANOVA with will used to compare groups using SAS software (Cary, NC). RESULTS/ANTICIPATED RESULTS: Feasibility studies show robust in vitro growth of patient-derived GBM cells, showing continued growth over 10 cellular division passages. Lentivirally transduced GFP reveals reliable tumor tracking both in vitro and in vivo. Transgenic mice at 6 months display reproducibly decreased pericyte and microvascular density in triplicate. Wild-type mice tolerate tumor injection up to three weeks with visible tumor growth, peritumoral hypervascularity, and no evidence of mouse neural dysfunction. With current cohorts recently implanted with tumor, we anticipate a significant decrease in tumor size with Cohen’s *d* effect size of 0.5 in GBM implanted in pericyte-deficient mice when compared to control. Effect sizes are based moderate to large (effect size 0.5–0.8) reductions of in vitro GBM growth in vascular gene (TGF-β knockdown studies). In addition, tagged tumor-derived pericytes should comprise a greater proportion of new vasculature in pericyte-knockdown mice to overcome host pericyte depletion. Finally, tumors in transgenic mice should show increased hypoxia from limitations in angiogenesis. DISCUSSION/SIGNIFICANCE OF IMPACT: Feasibility studies show successful tracking of fluorescently tagged-patient derived GBM samples in transgenic mice with decreased vasculature. GBM grafts show no evidence of immunogenic response with cyclosporine protocol. Successful limitation of tumor size with reduced pericyte density will provide support to increasing study of blood-brain barrier, stem cell activity and inflammatory activity of pericyte microenvironments altering GBM behavior. Furthermore, implementation of known pericyte targeted therapies, such as imantinib, can be evaluated for GBM patient treatment efficacy. Studies with assembled clinical translational research scholar mentorship team will allow the principal investigator to develop an independent career with laboratory focused on contributing to improved patient outcomes, translating successful pericyte-targeted results to patient trials.

